# Supplementary material for: Focal cortical seizures start as standing waves and propagate respecting homotopic connectivity
Source: Nat Commun. 2017 Aug 9;8:217. doi: 10.1038/s41467-017-00159-6 (PMC5550430; doi:10.1038/s41467-017-00159-6)
Supplement: Supplementary file 1 — Supplementary Information [file 41467_2017_159_MOESM1_ESM.pdf]

File Name: Supplementary Information
Descriptions: Supplementary Figures

File Name: Peer Review File
Descriptions:

File Name: Supplementary Movie 1
Descriptions: Example interictal events from 3 example animals. Leftmost panels: the retinotopic maps for each example animal. Right panels: 4 representative interictal events for each animal. The frame rate is 35 frames/s, two times slower compared to real time. Each event was normalized to its maximum ΔF/F value.

File Name: Supplementary Movie 2
Descriptions: Example seizures from 3 example animals. Left column: retinotopic maps for each example animal. Remaining panels: 4 representative seizures for each animal. The frame rate is 35 frames/s, 2 times slower compared to real time. Each event was normalized to its maximum ΔF/F value.

File Name: Supplementary Movie 3
Descriptions: Hilbert analysis of propagation of 6-11Hz oscillations from the example seizure in Figure 6 a,b and Supplementary Figure 8. Top left panel: GCaMP6f ΔF/F0 movie. Top right panel: 6-11Hz band pass filtered GCaMP6f signal. Bottom left panel: Hilbert amplitude of 6-11Hz oscillations during the same seizure. Bottom right panel: Hilbert phase of 6-11Hz oscillations for the same seizure. For each pixel, the color indicates the phase of the oscillation, while the saturation is proportional to the amplitude of the oscillation. For this panel only, the saturation was clipped at the 70th percentile of the maximum amplitude for display purposes.

File Name: Supplementary Movie 4
Descriptions: Example cycle average of 6-11 Hz oscillations during one seizure. Left panel: cycle average of the filtered 6-11Hz GCaMP oscillation, normalized to maximum and with retinotopic map superimposed. Right panel: cycle average of the Hilbert transform phase of the oscillation. The saturation indicates the amplitude of the oscillation for each pixel.

Supplementary figures

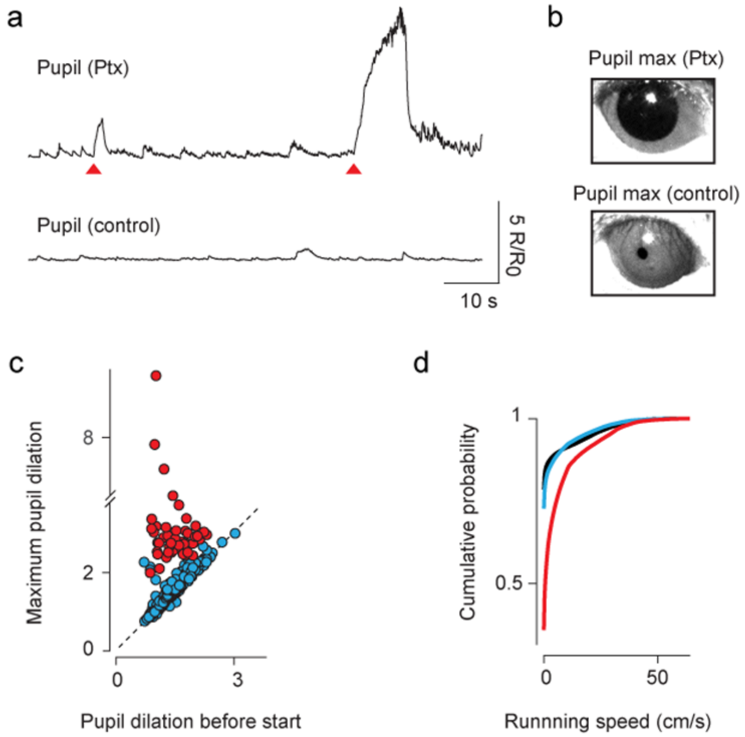

Supplementary Figure 1. Behavioral correlates of epileptiform events. (**a**) *Top panel:* Example pupil dilation recording during Ptx-triggered seizures in the visual cortex. *Red triangles* indicate seizure initiation. *Bottom panel*: Example recording of pupil radius in control physiological conditions, during the presentation of visual stimuli used for retinotopic mapping. (**b**) Comparison between maximum pupil dilations measured in control physiological conditions (*bottom panel*) versus maximum pupil dilation recorded during Ptx-triggered seizures (*top panel*), for the same example mice of panel **a**. Difference between maximum dilations in these two conditions was significant across mice ($p < 0.05$, two samples t-test, $n=3$). (**c**) Relationship between pupil dilation at the start (abscissa) and at maximum reached during epileptic events (ordinate) of the two types (**446** interictal events and **53** seizures from 3 animals). Dilations are measured relative to baseline pupil radius (R_0). (**b**) Cumulative distribution of running speed in a 2s window following the onset of interictal events (blue) and during seizures (orange), compared to baseline measurements obtained between events (black). Data normalized and averaged across **5 a**nimals.

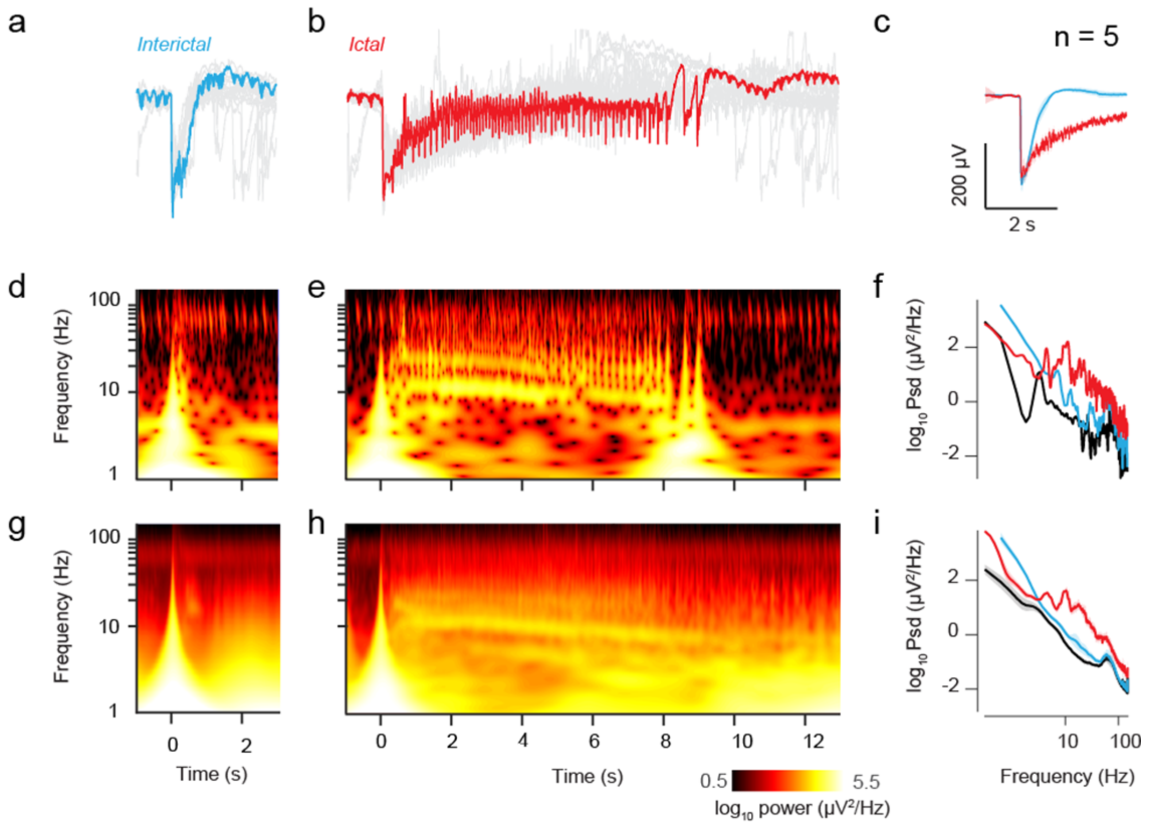

Supplementary Figure 2. Temporal and spectral signatures of interictal events and seizures measured from the LFP. (**a**) LFP waveform of a representative interictal event (blue) and of other example events recorded during the same experiment (gray). (**b**) Same as **a**, for seizures in the same experiment. (**c**) Average across animals (n = 5) of the LFP waveform following the onset of interictal events (blue) and seizures (orange). (**d**-**e**) Spectrograms (Morlet-wavelet scalograms) of the representative traces in **a** and **b**. (**f**) Power spectra of those two events measured over 3 s from onset, and compared to a representative baseline period (black). (**g**-**h**) Average spectrogram of interictal events (n = 1476) and seizures (n = 137), across 5 animals. (**i**) Average power spectrum of the events, obtained by averaging curves such as those in **f** across events and animals. The width of the curves indicates 2 s.e.m.

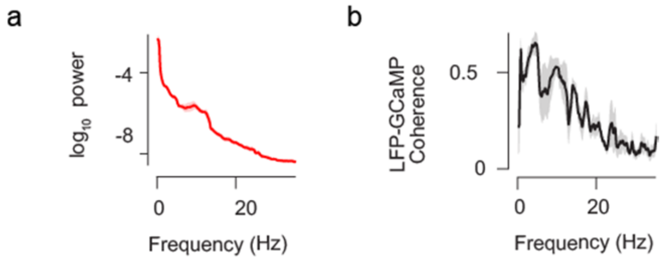

Supplementary Figure 3. Frequency content of GCaMP signals. (a) Average power spectrum of the GCaMP signals recorded over the visual cortex during seizures, across animals (n = 5). (b) Average coherence between the LFP and the GCaMP signals recorded during seizures across animals (n = 5).

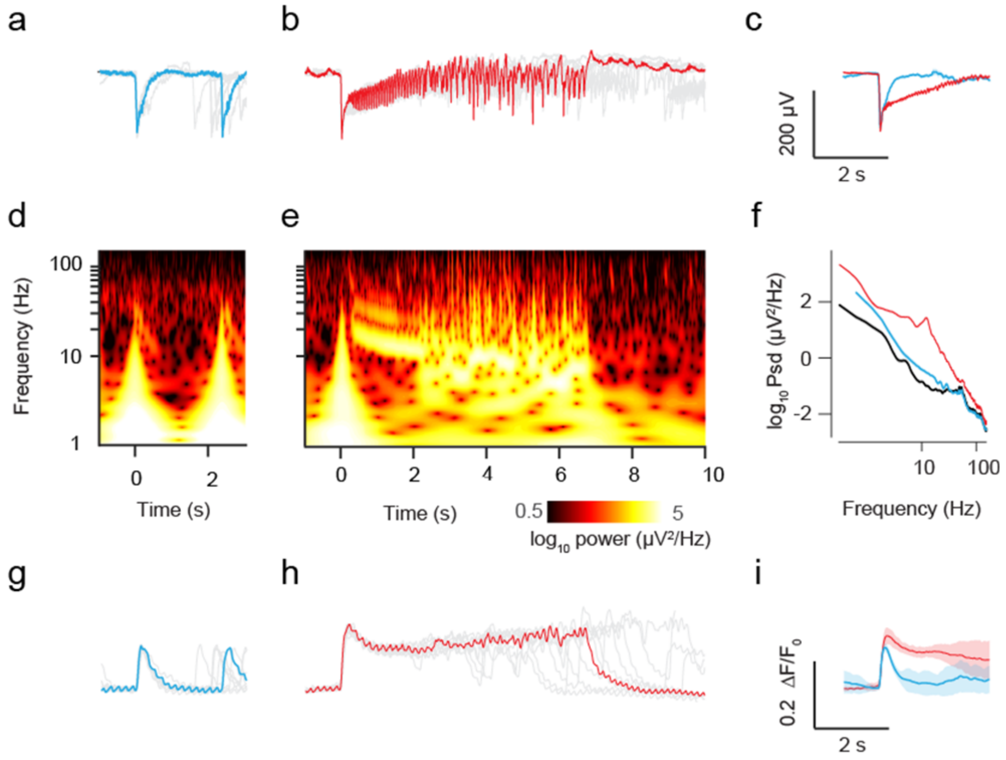

Supplementary Figure 4. Pilocarpine-induced epileptic focus recapitulates results obtained with the picrotoxin model. (**a**) LFP waveform of a representative interictal event (blue) and of other example events recorded during the same experiment (gray). (**b**) Same as **a**, for seizures in the same experiment. (**c**) Average of the LFP waveform from one animal following the onset of interictal events (blue) and seizures (orange). (**d**-**e**) Spectrograms (Morlet-wavelet scalograms) of the representative traces in **a** and **b**. (**f**) Average power spectrum of interictal events and seizures for one animal, compared to power spectrum of baseline activity. (**g**) GCaMP activity averaged over visual cortex during the example interictal events in **a**. (**h**) Same as **g**, for the seizures shown in **b**.)(**i**) Average of GCaMP fluorescence averaged over the visual cortex from one animal following the onset of interictal events (blue) and seizures (orange) (**j**) Variance explained by the standing wave model for 1 s sections of interictal events (*blue dot*) and seizures (*red dots*). Error bars show median ± 1 quartile. Shaded blue area indicates the 96% confidence interval for quality of the fit to interictal events. (**k**) Comparison between maximum signal recorded in picrotoxin (n=3) and pilocarpine (n=2) experiments, including GCaMP3 animals only, during interictal events or seizures (** = p<0.001, * p = <0.05, two samples t-test). (**l**) Comparison between the area of the epileptic focus induced in picrotoxin (n=3) and pilocarpine (n=2) experiments, including GCaMP3 animals only (ns = no significant difference found, two sample t-test).

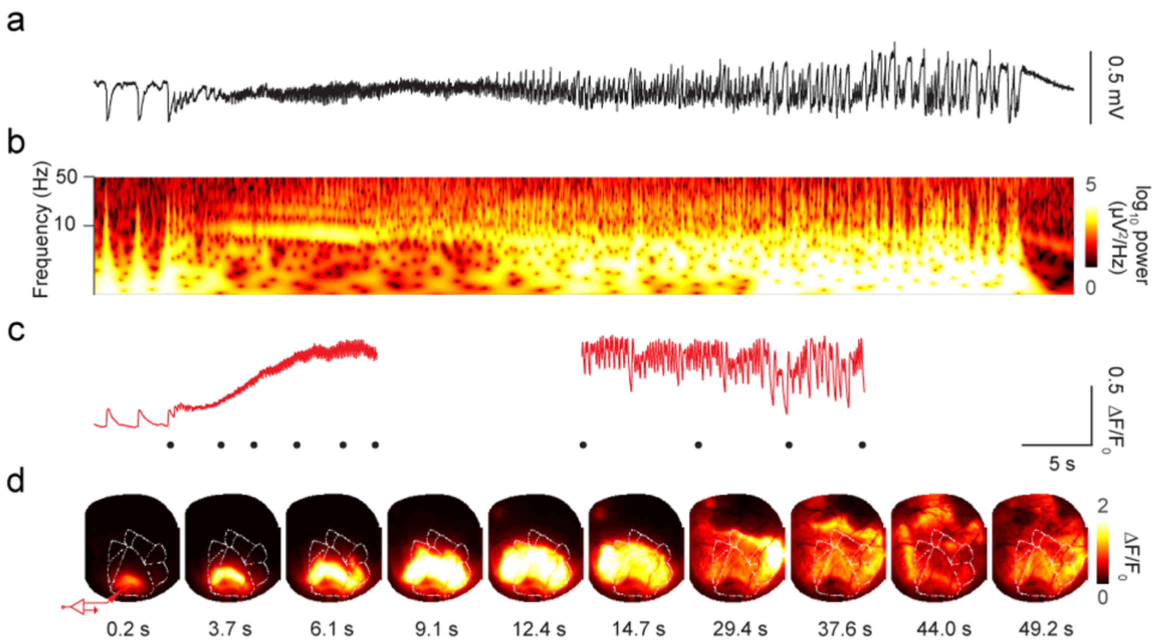

Supplementary Figure 5. Prolonged seizures invade the whole imaged hemisphere. (**a**) LFP recording from the focus. (**b**) Morlet-wavelet spectrogram of the recorded LFP. (**c**) GCaMP6f signal averaged across the whole imaging window, aligned to the LFP (**d**) Single frame snippets from the GCaMP movie, corresponding the black dots in panel **c.** The red cartoon electrode indicates the site of Ptx injection and LFP recording.

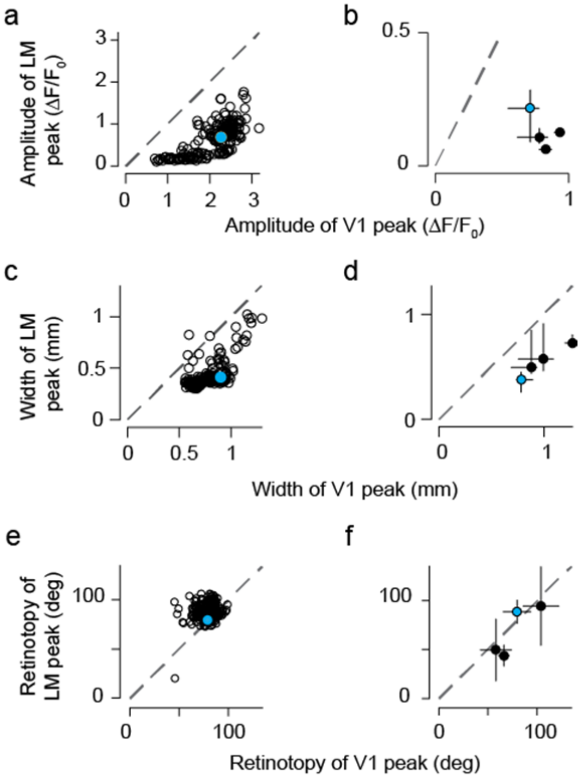

Supplementary Figure 6. Characterizing the two homotopic responses during interictal events. (**a-f**) Response profiles from Figure4 were fit with two Gaussians. The left panels (**a**, **c**, **e**) summarize the fit for each event from the example animal in Figure 4; the right panels (**b**, **d**, **f**) show the data from every animal, median ± first quartile. (**a-b**) The peak response in V1 is plotted against the peak response in LM. To allow comparison between different GCaMP variant, data where normalized to the max V1 response in the right panel. (**c-d**) The standard deviation of the Gaussian fit in V1 is plotted against the standard deviation of the fit for LM. (**e-f**) The retinotopic position of the fit in V1 is plotted against the retinotopic position of the peak in LM.

Supplementary Figure 7. Selection of pixels recruited into the seizure. (**a**) Distribution of maximum pixel intensities during seizures for one animal (n= 14), normalized to absolute maximum. Black dot's shows the average distribution, gray dots single trial distribution. Red line is a double Gaussian fit to the average data, individually normalized to absolute maximum. (**b**) Average distribution of maximum pixel intensities during seizures for 4 animals. On average, a threshold of 0.3 (dashed line) separates well the population of pixels with greater activity.

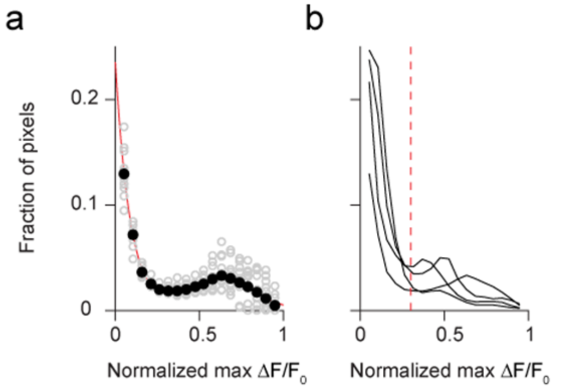

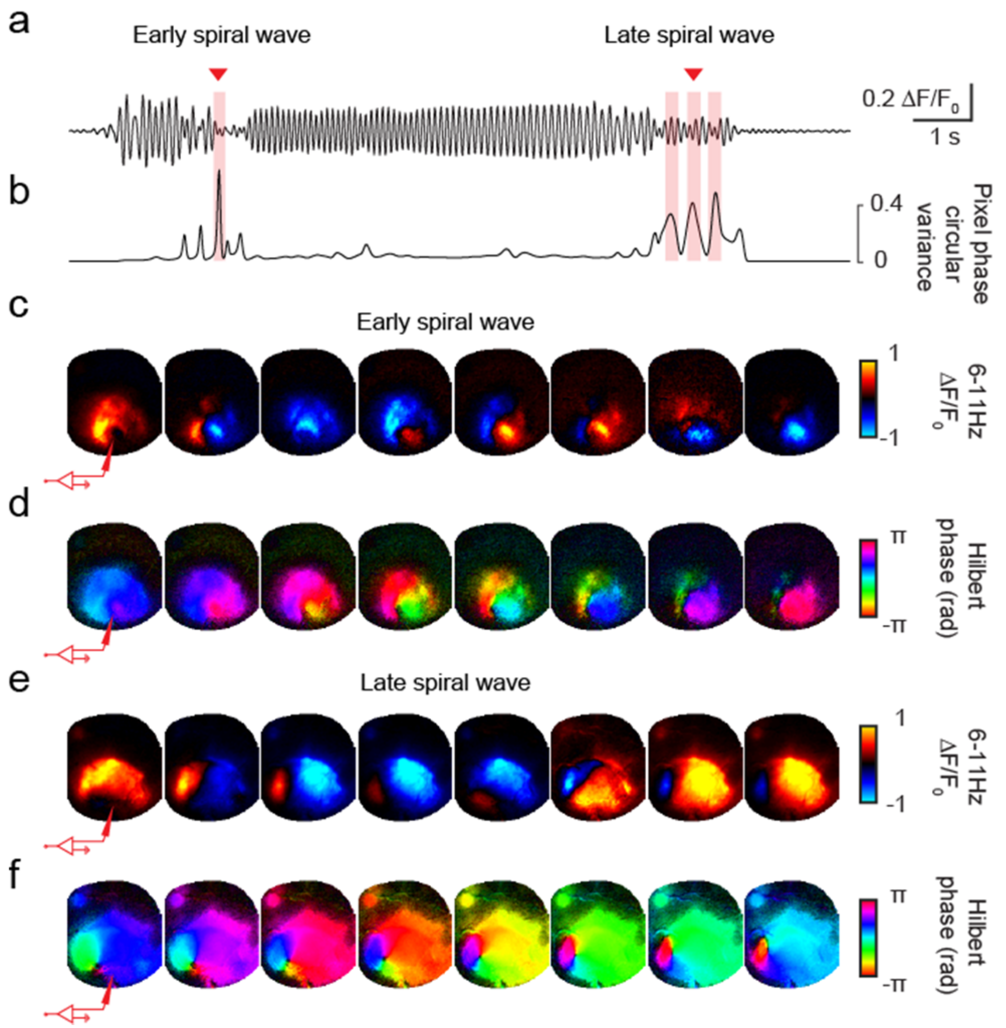

Supplementary Figure 8. Spiral waves during seizures. (**a**) Average GCaMP6 signal from the focus during a seizure, band-pass filtered between 6 and 11 Hz. Shaded boxes highlight cycles when spiral waves were recorded. (**b**) Time course of circular variance of the phases for pixels with significant oscillation amplitude, computed across each frame. (**c**) Example oscillation cycle showing the propagation of an early spiral wave (highlighted in **a**). For each frame, pixels were normalized to the maximum absolute value for visualization purpose. (**d**) Hilbert transform representation of the example early spiral wave from panel **c**. For each pixel, hue represent the phase, while saturation represent amplitude of the oscillation. (**e, f**) Same as in **c, d** for the late spiral wave highlighted in **a**. In both spiral waves examples, note the calcium activity revolving around a pinwheel with reduced oscillation amplitude. The cartoon electrode indicates the location of the LFP pipette.
